# The current status of recommendations for non-invasive neuromodulation therapy in severe mental disorders

**DOI:** 10.1192/j.eurpsy.2024.1467

**Published:** 2024-08-27

**Authors:** O. Vasiliu

**Affiliations:** Psychiatry, Dr. Carol Davila University Emergency Central Military Hospital, Bucharest, Romania

## Abstract

**Introduction:**

There is an increasing rate of treatment resistance in severe psychiatric disorders (SPDs), which indicates the necessity for finding new therapeutic interventions, because of the significant negative impact these disorders have on the patient’s quality of life, functionality, and other important parameters. In clinical practice, SPDs are estimated to represent up to 30-60% of all diagnosed cases. Schizophrenia spectrum disorders (SSD), major depressive disorder (MDD), and bipolar disorders (BDs) are associated with lower response to a large variety of therapeutic approaches. In this context, new technologies should be considered for SPDs, and non-invasive neuromodulation techniques can be explored as add-ons to ongoing therapeutic interventions.

**Objectives:**

A literature review was conducted to detect the available evidence to support recommendations for neuromodulation techniques in SPDs.

**Methods:**

Three electronic databases (PubMed, Cochrane, Google Scholar) were searched for papers corresponding to the keywords “treatment-resistant psychiatric disorders” and “neuromodulation” or “electroconvulsive therapy” (ECT) or “transcranial magnetic stimulation” (TMS) or “transcranial direct current stimulation” (tDCS), published from the beginning of the respective databases up to July 2023.

**Results:**

After the initial search, 1258 papers surfaced, but only 72 remained to be included in the analysis, after filtering them according to the inclusion and exclusion criteria. TMS may improve both depressive and manic symptoms, but also reports of polarity changes were found, indicating the need for careful monitoring of treatment-emergent affective switches (TEAS). TMS may also improve cognitive functions, although not sufficient evidence was found to support this observation clearly. The efficacy of temporoparietal TMS in schizophrenia has not been proven with certainty, although this intervention may improve positive symptoms. ECT was an effective and well-tolerated intervention for severe mood episodes, SSD, and BDs. Depressive symptoms responded to tDCS in bipolar/monopolar patients, but reports of TEAS in the BDs population have been reported.

**Conclusions:**

Non-invasive neuromodulation techniques may represent an efficient option in patients with SPD, but more good-quality trials are needed before this recommendation is formulated in clinical guidelines.

**Disclosure of Interest:**

None Declared

